# Physiology of Cerebellar Reserve: Redundancy and Plasticity of a Modular Machine

**DOI:** 10.3390/ijms22094777

**Published:** 2021-04-30

**Authors:** Hiroshi Mitoma, Shinji Kakei, Kazuhiko Yamaguchi, Mario Manto

**Affiliations:** 1 Department of Medical Education, Tokyo Medical University, Tokyo 160-8402, Japan; 2 Laboratory for Movement Disorders, Tokyo Metropolitan Institute of Medical Science, Tokyo 156-8506, Japan; kakei-sj@igakuken.or.jp; 3 Department of Ultrastructural Research, National Center of Neurology and Psychiatry, National Institute of Neuroscience, Tokyo 187-8551, Japan; rkmayamaguchi@yahoo.co.jp; 4 Unité des Ataxies Cérébelleuses, Service de Neurologie, Médiathèque Jean Jacquy, CHU-Charleroi, 6000 Charleroi, Belgium; mmanto@ulb.ac.be; 5 Service des Neurosciences, University of Mons, 7000 Mons, Belgium

**Keywords:** cerebellar reserve, cerebellar ataxias, synaptic plasticity, long-term depression

## Abstract

The cerebellum is endowed with the capacity for compensation and restoration after pathological injury, a property known as cerebellar reserve. Such capacity is attributed to two unique morphological and physiological features of the cerebellum. First, mossy fibers that convey peripheral and central information run mediolaterally over a wide area of the cerebellum, resulting in the innervation of multiple microzones, commonly known as cerebellar functional units. Thus, a single microzone receives redundant information that can be used in pathological conditions. Secondly, the circuitry is characterized by a co-operative interplay among various forms of synaptic plasticity. Recent progress in understanding the mechanisms of redundant information and synaptic plasticity has allowed outlining therapeutic strategies potentiating these neural substrates to enhance the cerebellar reserve, taking advantage of the unique physiological properties of the cerebellum which appears as a modular and potentially reconfiguring brain structure.

## 1. Definition of Cerebellar Reserve

Cerebellar reserve is defined as the capacity for tissue compensation and restoration following pathological injury [1,2,3]. This concept dates back to more than a century ago and appeared in a classical clinical monograph by Holmes (pp. 514–515) [4]. One patient developed a lesion limited to the lateral lobe of the cerebellum, with cerebellar ataxia (CA) disappearing spontaneously after 58 days. Another patient with a larger lesion in the lateral and medial cerebellar lobes presented with a more severe CA, which later showed complete resolution after 71 days. Notably, there was no difference in the gait between these two patients during the recovery phase, despite the marked difference in the extent of the two lesions.

Depending on the nature of the etiology, two types of cerebellar compensation and restoration can develop [3]. When the etiology elicits a transient structural damage in a limited area (e.g., in cases of stroke and traumatic injury), the lost cerebellar functions can be compensated for by other cerebellar areas not affected by the structural loss. On the other hand, when the etiology weakens cerebellar neurons and glial cells in diffuse areas, gradually leading to cell death (e.g., in case of immune-mediated, metabolic, and degenerative CAs), vanishing cerebellar functions are replenished by the affected lesion itself through functional reorganization. We like to term the former type structural cerebellar reserve and the latter type functional cerebellar reserve [3].

The concept of reserve was proposed initially to account for differences in susceptibility of the cerebral cortex to the aging process and pathological damage [5,6,7,8]. Here, we define reserve as a moderator between pathology and outcome [5]. The tolerance to aging/pathology is in part attributed to morphological and quantitative features, such as the number of remaining intact/undamaged neurons and synapses [5,6,7]. Alternatively, the resilience is linked to the extent of functional activity, e.g., the utilization of pre-existing cognitive storage or the integrity of functional brain networks [5,6,7]. Besides, in Parkinson’s disease (PD), an individual’s capacity to react to the pathology is also variable [9]. It was recently shown that functional connections within the motor reserve network are associated with the individual’s capacity to cope with PD-related pathologies [9]. However, it is still unclear what components in the cerebral/basal ganglia circuits and synapses constitute the reserve.

In contrast, cerebellar functions have been defined at the levels of neural circuits and synapses in the last five decades [10]. The brain comprises 85–100 billion neurons, and about 60% of these neurons are located in the cerebellum, although the cerebellum constitutes only about 10% of the brain mass [11,12]. Recent uncovering of the underlying mechanisms and network theories provide clues to the understanding of fundamental mechanisms underlying dynamic reorganization. The present review focuses on two neural substrates that can actively cope with various types of cerebellar pathologies. First, we show that the functional unit in the cerebellar cortex, termed the microzone, receives various central and periphery inputs and that such redundant information can be utilized in pathological conditions [3]. Second, we review the cooperation among various forms of synaptic plasticity [13]. Finally, we propose possible strategies that can potentiate these neural substrates to enhance the cerebellar reserve.

## 2. Redundant Input Organization in the Cerebro–Cerebellum

### 2.1. Neural Substrate for Internal Forward Model

State prediction with a forward model is a neural mechanism that allows rapid and stable control of movement, even when peripheral sensory feedback has a temporal delay [14,15]. This predictive computation is known as an internal forward model and considered to be located in the cerebellar circuit. The most important requirement of a forward model in motor control is the integration of various inputs to predict the state of the future motor apparatus for a motor command. More specifically, a forward model needs the integration of two lines of essential inputs: (1) an efference copy (copy of a motor command) from the controller; (2) a sensory feedback signal that describes slightly past (~100 ms) state of the motor apparatus [14,15].

#### 2.1.1. Efference Copies 

**Figure 1:**
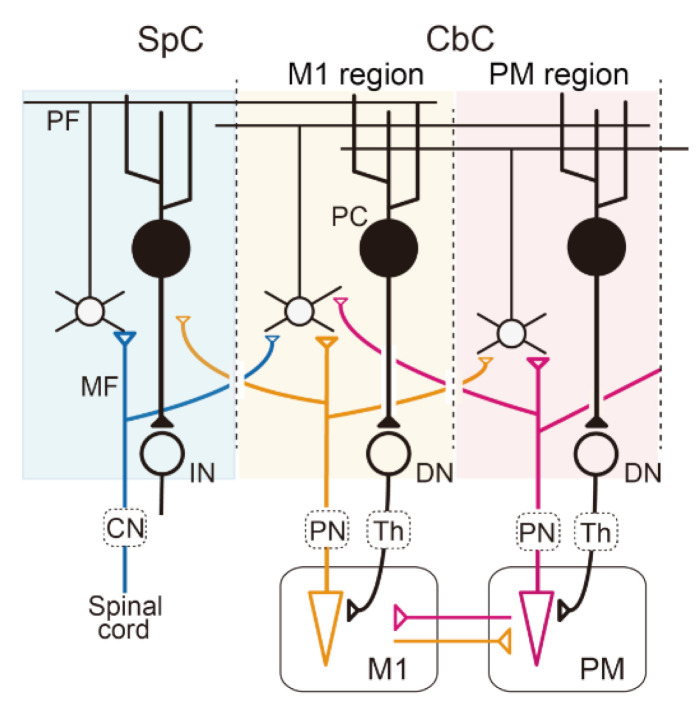
Multiple communication loops with extensive overlaps A: Schematic diagram of the cerebellar circuit. Mossy fiber (MF) inputs from the periphery, the primary motor cortex (M1), and the premotor cortex (PM) are represented by blue, orange, and red lines, respectively. In this diagram, we assume that the sensory afferent and efference copies from the M1are integrated into individual GCs or PCs in the M1 region of the cerebro–cerebellum (CbC). The output from the M1 region of the CbC projects back to the M1 through the dentate nucleus (DN) and the thalamus (Th). CbC, cerebro–cerebellum; CN, cuneate nucleus; IN, interpositus nucleus; PC, Purkinje cells; PN, pontine nuclei; SpC, spino–cerebellum.

Given that the motor command is generated in the primary motor cortex (M1), it is likely that the region of the cerebro–cerebellum that receives M1 inputs serves as a forward model. In general, the cerebro–cerebellum receives its primary input through the cortico–ponto–cerebellar pathway (Figure 1), and layer V corticofugal neurons in M1 send inputs to the pontine nuclei (Figure 1, PN) [16]. Therefore, the region of the cerebro–cerebellum is presumed to receive an efference copy of the motor command through the pathway and monitors the current motor command on line with minimum delay (very probably less than 10 ms due to the distance and velocity of propagation of action potentials through myelinated axons). However, only a few studies have investigated the activities of the ponto–cerebellar mossy fiber (MFs) (Figure 1, MF) projections in the cerebro–cerebellum during voluntary limb movements. By definition, the efference copy inputs are assumed to show movement-related activities that lag little behind those of M1 neurons. Van Kan et al. (1993) demonstrated that MFs in the intermediate part of the cerebellum in monkeys were active during limb movement and that the onset of the activity preceded the onset of movement in the majority of MFs (the mean lead time was about 80 ms) [17]. Recently, we reported similar movement-related MF activities in the cerebro–cerebellum for wrist movements [18,19]. In that experiment, monkeys were trained to perform step-tracking wrist movements in eight evenly spaced directions, and we analyzed the task-related activities of MFs in the hemispheric parts of lobules V and VI, which are the most strongly connected with M1 [20,21]. We found modulation of the activity of the majority of MFs before movement onset, and the modulation lagged slightly behind that of M1 neurons in the same experimental setup [22]. In addition, we also found that directional tuning of those MFs demonstrated a significant shift in the preferred direction (PD) for different forearm postures [19] just like muscle-like neurons in M1 [22]. Therefore, the activity of the MFs appears to represent intrinsic (i.e., somatic) information rather than extrinsic information. Overall, it is most likely that the MF inputs to the cerebro–cerebellum convey an efference copy of motor commands (Figure 1).

The slight lag of the MF modulation relative to that of M1 neurons almost excludes the likelihood that the region of the cerebro–cerebellum serves as an inverse model for M1. On the other hand, MF inputs that encode extrinsic information may be represented heavily in the region of the cerebro–cerebellum that is more lateral to the M1 region, at the projection of the premotor area (PM), which represents spatial or visual information of movement [23,24]. It should be also noted that this region does not comprise part of the inverse model for M1, because its output does not return to M1, but to PM (Figure 1) [20,21,24].

#### 2.1.2. Sensory Feedback Signals 

Forward models also require sensory feedback signals from the periphery that provide the current state of the body. Indeed, the cerebellum receives strong proprioceptive and cutaneous afferents directly through the cuneocerebellar and rostral spinocerebellar tracts from the arm and through the dorsal and ventral spinocerebellar tracts from the leg in cats [25,26,27]. These afferents terminate as MFs in lobules IV and V of the intermediate zone of the cerebellum (summarized in Ito 1984) [28]. Although detailed experiments on these pathways have not been conducted in primates, it is plausible to presume that primates also have the same sensory pathway to the cerebellum (Figure 1). The somatosensory inputs should enable the cerebellum to monitor the current state of the body with minimal delay. According to Jörntell and Ekerot (2006), electrical skin stimulation evokes the excitation of granule cells (GCs) in no more than 6–8 ms in decerebrated cats [29]. Indeed, in conscious monkeys, we confirmed that most MFs in the hemispheric part of lobules V and VI responded vigorously to manual somatosensory stimuli, such as gentle palpation of muscles, extension/flexion of joints, and light touch of the skin [30]. In addition, the cerebro–ponto–cerebellar input from the primary somatosensory cortex (S1) [31] may provide another path for the somatosensory input to the cerebro–cerebellum in monkeys. Furthermore, MFs derived from M1 may also provide somatosensory inputs to the cerebro–cerebellum, because almost all M1 and PM neurons are strongly responsive to somatosensory stimuli [22,23].

In either case, the cerebro–cerebellum that forms a loop connection between cortical motor areas appears to receive both the efference copy and the somatosensory inputs required for a neuronal substrate to serve as a forward model.

### 2.2. Multimodalities and Loosely Organized Somatotopic Organization

In a forward model, motor and sensory inputs must be integrated to construct an output based on combinations of those inputs. There are morphologic substrates for the integration. The branching patterns of individual MFs are intensively divergent especially along the medio-lateral axis [32,33,34,35,36], despite the largely topographic projection of the MF inputs to the cerebellar cortex [20,21,24,37,38]. In other words, MF inputs are highly convergent to each microzone of the cerebellar cortex (see also Jörntell and Ekerot, 2002) [34]. Indeed, Huang et al. (2013) recently demonstrated the convergence of inputs from the basilar pontine nucleus (BPN) and the external cuneate nucleus onto individual GCs in the paramedian lobule [39]. They also demonstrated that BPN neurons projecting to the paramedian lobule receive inputs from M1. These lines of evidence demonstrate that efference copies and somatosensory afferent inputs are indeed integrated on single GCs in the cerebro–cerebellum. A huge number of GCs provide an even larger number of combinations of different sources of MF inputs.

Integration of those different lines of inputs proceeds even further on Purkinje cells (PCs), because (1) outputs of GCs (i.e., parallel fibers (PFs)) run more than several millimeters mediolaterally along folia of the cerebellar cortex, and (2) each PC receives inputs from numerous (>>10^4^) PFs (summarized in Ito 1984) [28]. Indeed, almost all PCs that show pre-movement modulation, which presumably originates from M1, are highly responsive to somatosensory stimuli [19,30]. In other words, these PCs in the cerebro–cerebellum are multimodal in the sense that they integrate motor as well as sensory inputs. Overall, the input organization of the cerebro–cerebellum comprises a huge amount of redundancy, which provides plenty of room for reorganization.

It is important to note that receptive fields (RFs) of the wrist-movement-related MFs and PCs are confined to a small part of the forearm [30] and are not responsive to stimuli in other body parts, such as face, trunk, or legs. In contrast, non-wrist-related neurons that are active for movements of other body parts (such as the leg or trunk) have RFs in the corresponding parts of the body. These cells are topographically organized, and therefore, one can assume the presence of certain somatotopic organization in the cerebellar cortex. It is important to emphasize that cerebellar neurons with different RFs are distributed over the cerebellar cortex with a considerable overlap (i.e., intermingled) [19,30]; namely, there are no sharp border lines between different somatotopic regions. The loosely organized somatotopic organization was already demonstrated by morphologic [21] and physiologic [40,41] studies and provides another evidence of “redundant input organization in the cerebro–cerebellum”.

### 2.3. Combinatorial Code with IO Inputs and Redundant MF Inputs

**Figure 2:**
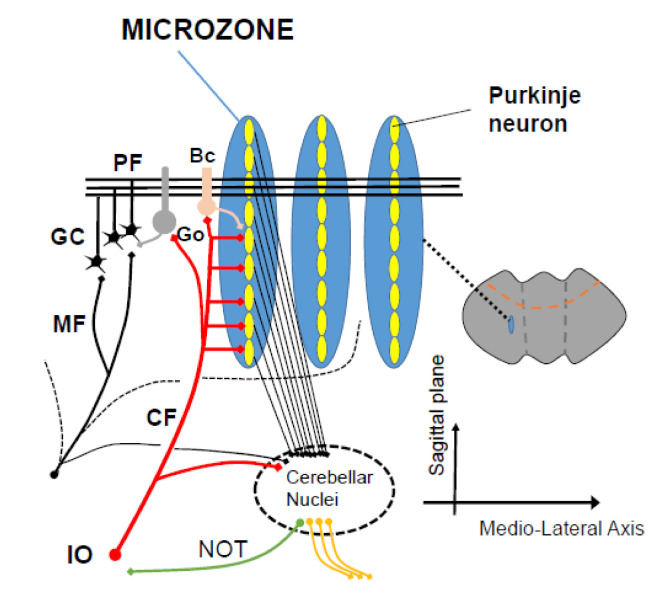
A scheme of microzones. A functional congruence between the 2 major input systems (mossy fibers, climbing fibers) is observed anatomically, with a contribution of mossy fibers into multizonal microcomplexes integrated in cerebellar modules subserving the operational aspects of the cerebellar machinery. PF: parallel fiber, CF: climbing fiber, GC granule cell, Go: Golgi cells, Bc: basket cell, IO: inferior olive.

PFs intersect the sagittally oriented dendritic trees of PCs, whereas the climbing fiber (CF) inputs enter the cerebellar cortex perpendicularly to the PFs [42,43,44,45] (Figure 2). About 10 to 15 PCs arranged in a sagittal microzone receive the same CF input [46]. Therefore, the PF inputs (i.e., MF inputs) are combined with the signal carried by the CF inputs. Each microzone integrates PF inputs depending on CF inputs so as to send specifically reorganized outputs to deep cerebellar nucleus (DCN) neurons [42,43,44,45]. Thus, the combinatorial code with the redundant MF inputs and IO inputs may provide potential functional flexibility to generate reorganized outputs for altered MF and/or CF inputs.

## 3. Multiple Forms of Synaptic Plasticity in the Cerebellum

**Figure 3:**
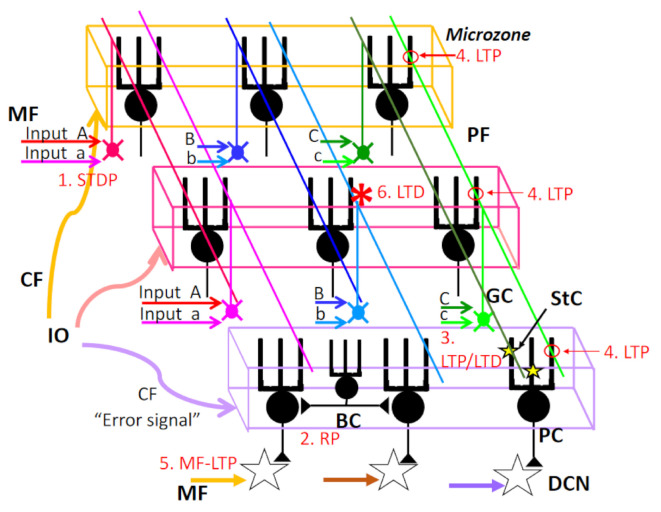
Multiple forms of synaptic plasticity with a special concern of spatial range. (1) STDP: spike timing-dependent plasticity MF–GrC synapse. (2) RP: rebound potentiation at inhibitory interneuron–PC synapse. RP should be induced in PCs innervated by the same CF (microzone). (3) LTP/LTD: Long-term potentiation or long-term depression at StC–PC synapses. This type of plasticity should be part of PC dendrites. (4) LTP: LTP at GrC–PC synapse. The postsynaptic type of LTP should be induced at PF–PC synapse along the same PF, except if conjunctively activated by CF. (5) MF-LTP: LTP at MF–DCN synapse. (6) LTD: LTD at PF–PC synapse. LTD should be induced at PF–PC synapses only when both PF–PC synapse and CF–PC synapse are activated conjunctively. See the text for further details. MF, mossy fiber; PF, parallel fiber; GC, granule cell; StC, stellate cell; PC, Purkinje cell; BC, basket cell; DCN, deep cerebellar nucleus neurons.

In the cerebellar cortex, multiple forms of synaptic plasticity at different sites are induced during procedural memory formation in the cerebellum (Figure 3) [47]. These multiple forms of synaptic plasticity seem to contribute to various aspects of the cerebellar reserve.

### 3.1. Spike Timing-Dependent Plasticity at Mossy Fiber–Granule Cell Synapses

At the cerebellum input stage, the existence of spike timing-dependent plasticity (STDP) between MF–GC synapses was reported previously (1. STDP in Figure 3) [48]. Spike timing-dependent long-term potentiation and depression (st-LTP and st-LTD) were confined to a ±25 ms time window. Since excitatory postsynaptic potentials (EPSPs) inducing action potentials (APs) cause st-LTP of EPSPs, while APs preceding to EPSPs cause st-LTD of EPSPs, the STDP is Hebbian in nature. EPSP–spike pairing at 6 Hz optimally induced STDP; thus, STDP can associate plasticity to the MF burst phase with high temporal precision.

As for MF inputs to the GCs, it is known that the input is a highly multimodal structure, receiving signals from various sensory modalities and integrating them during complex sensorimotor coordination tasks (see previous section). An in vivo patch-clamp study confirmed that a subset of single GC receive convergent functional multimodal (somatosensory, auditory, and visual) inputs via separate MFs. Furthermore, the integration of multimodal signals enhances the probability of action potential generation of GCs [49]. A set of multimodal input converges on GC with adequate timing, leading to the st-LTP of STDP. This type of synaptic plasticity serves as an amplifier of cerebellar inputs. During the process of compensatory learning following damage of the internal model, some of the new set of multimodal sensory input and efferent copy of cerebral command may be enhanced by STDP at the input stage of cerebellar cortex.

### 3.2. Rebound Potentiation of Inhibitory Inputs to Purkinje Cells

With regard to the synaptic plasticity of inhibitory inputs to PCs, GABA-mediated inhibitory synaptic transmission undergoes long-lasting “rebound potentiation (RP)” after repetitive activation of excitatory CF inputs (2. RP in Figure 3) [50] or repetitive somatic depolarization. Both processes cause Ca^2+^ influx through voltage-gated Ca^2+^ channels and activate Ca^2+^/calmodulin-dependent protein kinase II (CaMKII), which causes structural alteration of GABA_A_R-associated protein (GABARAP) and subsequently enhances the interaction between GABARAP and GABA_A_R γ2 subunits, leading to an increase in GABA_A_R expression at the inhibitory synaptic sites [51].

In transgenic mice expressing an inhibitory peptide that blocked the interaction between GABARAP and GABA_A_R, RP was selectively impaired, and VOR adaptation was lost, but not OKR adaptation [52]. Thus, RP is assumed to contribute to cerebellar learning with instructor, since CFs convey the error signal [53].

The direction of alternation of PC activity is the same as that of PF-PC LTD, namely, CF activity-dependent decrease in PC activity. Although the spatial distribution of RP in the cerebellar cortex in vivo is unknown, RP would be elicited in a large number of PCs belonging to the same microzone, in which PCs are innervated by the same CF [54] (Figure 2). Thus, RP provides learning with more coarse “spatial resolution” than that of PF-PC LTD. Based on the spatial distribution, we hypothesize that coarse learning by RP is necessary for subsequent fine learning by LTD. This hypothesis explains the reason for the failure of VOR adaptation in RP-deficient but LTD-intact animal [52].

### 3.3. LTP/LTD at Parallel Fiber–Stellate Cell Synapses

Synaptic plasticity was reported also at the synapse between PF and stellate cell (StC), a molecular layer inhibitory neuron (3. LTP/LTD in Figure 3). Repetitive stimulation of PF elicits the postsynaptic type of LTP or LTD in StC [55]. The relationship between these types of synaptic plasticity and learned behavior is elusive. However, the resolution in learning processes would be intermediate between RP and LTD, because the ratio of the molecular layer interneurons (StC + Basket cell) vs. PC is reported to be around 5 [56].

### 3.4. LTP at Parallel Fiber–Purkinje Cell Synapse

PF-LTP is induced by PF activity alone. Two types of PF-LTP; presynaptic PF-LTP and postsynaptic PF-LTP, have been reported (4. LTP in Figure 3). The presynaptic LTP, namely, an increase in neurotransmitter release from the PF terminal, is induced by PF stimulation alone at 4–8 Hz and requires cAMP-dependent phosphorylation of active zone protein RIM1α [57,58,59]. In contrast, PF stimulation alone at 1 Hz causes postsynaptic PF-LTP, in which the intracellular concentration of Ca^2+^ transiently increases to a level lower than that observed following induction of PF-LTD. Such an increase in Ca^2+^ activates PP2B (Calcineurin), which results in dephosphorylation of AMPA receptors [60,61] with subsequent stabilization of AMPAR at the synaptic membrane through interaction with scaffold proteins. Furthermore, postsynaptic PF-LTP depends on nitric oxide (NO), which modulates N-ethylmaleimide-sensitive fusion (NSF) protein, an ATPase. NSF, activated by S-nitrosylation, binds the AP-2/NSF site of GluA2-CT, leading to enhanced synaptic insertion of GluA2-containing AMPAR via exocytosis [62,63].

Purkinje cell-selective deletion of PP2B in mice abolished postsynaptic PF-LTP, whereas LTD was unaffected. The same mutant mice showed impaired “gain-decrease” and “gain-increase” adaptation of the vestibulo-ocular reflex (VOR) as well as impaired acquisition of classical delay conditioning of the eyeblink response [61], suggesting the specific role of PF-LTP in cerebellar learning. The postsynaptic type of PF-LTP can reset PF-LTD, and, vice versa, PF-LTD can reset PF-LTP. In this regard, PF-LTP and PF-LTD mutually counterbalance each other [63]. In other words, PF-LTD would easily saturate, and learning of new internal model would be difficult if LTP does not counterbalances PF-LTD. On the other hand, the roles of presynaptic PF-LTP in cerebellar learning remain elusive.

### 3.5. Synaptic Plasticity at the Synapse between Mossy Fibers and Deep Cerebellar Nucleus Neurons

PCs project their axons to DCN neurons and strongly inhibit their activity. With regard to eye-blink conditioning, LTD at the PF–PC synapses is considered to play a key role in initial plasticity, but the basic essential memory trace is formed and stored in the anterior interpositus nucleus (IPN) for classical conditioning of the eye-blink response [64]. In agreement with this assumption, enhancement of excitatory postsynaptic currents (EPSCs) at MF–IPN synapses was induced using a protocol that mimics eye-blink conditioning in vitro (high-frequency MF stimulation and postsynaptic hyperpolarization) [65] (5. MF-LTP in Figure 3).

Similar to OKR adaptation, the functional memory trace of short-term adaptation (1 day) is formed initially within the cerebellar cortex and later transferred to the vestibular nuclei to be consolidated for long-term memory (week-long) [66]. The amplitude and slope of the evoked monosynaptic field response (N1) in week-long adapted mice were enhanced around the medial vestibular nucleus compared with those of control mice [66]. These MF-LTP in DCN neurons contribute to the compensatory learning as part of the cerebellar reserve, collaborating with learning in PCs.

Interestingly, enzymatic depletion of the perineuronal nets (PNNs), an extracellular matrix composed mainly of chondroitin sulfate proteoglycans, in DCN causes increase in the PC-IPSC amplitude recorded from DCN neurons in vitro, while enzymatic depletion of PNNs in IPN enhances the delay of eye-blink conditioning in vivo [67]. These results provide the background for the development of new therapies that can enhance cerebellar reserve.

### 3.6. Evidence for Involvement of Parallel Fiber–Purkinje Cell LTD in Motor Learning

The multiple forms of synaptic plasticity in the cerebellar cortex challenges the hypothesis that PF-LTD (6. LTD in Figure 3) is critically important for cerebellar motor learning. Actually, motor learning was normal in some mutant mice that apparently lacked PF-PC LTD, thus arguing against the importance of PF-LTD in motor learning [68].

However, recent experiments have provided substantial evidence for the key role of LTD in motor learning. First, the results of gene manipulations should be carefully interpreted. Generally, in gene-manipulated animals, unknown compensatory mechanisms of synaptic plasticity are possibly expressed, and the necessary conditions required for the induction of compensated synaptic plasticity might be different from ordinal experimental conditions that can otherwise induce LTP or LTD in the wild-type animal. Consistently, using various forms of LTD-inducing stimulation protocols, LTD could be successfully induced in the same PF–PC in LTD-lacking mutant mice [69], suggesting that the LTD hypothesis could not be ruled out. Second, using a new optogenetic blocker of endocytosis, LTD can be blocked reversibly. This tool (PhotonSABER) enables the temporal, spatial, and cell-type specific control of AMPA receptor endocytosis at active synapses. In one study in which LTD was blocked by photostimulation in vivo, VOR adaptation was impaired [70]. This photoactivation with PhotonSABER affected neither RB nor LTP. These results show that although RB and LTP were intact, LTD blockade impaired VOR adaptation. Thus, LTD seems necessary for cerebellar motor learning.

In conclusion, various types of synaptic plasticity (STDP, RP, Stc-PC LTP/LTD, PF-PC LTP, and MF-LTP) in the cerebral cortex seem to contribute to motor learning through an increase in the contrast of input signals, efficiency and accuracy of learning, and storage of learned engram. Collaborating with subordinate synaptic plasticity in the cerebellum, PF–PC LTD could play a crucial role in cerebellar learning by adjusting the final stage of cerebellar–cortical integration.

### 3.7. Improvement of Symptoms and Synaptic Plasticity

Even in patients with degenerative cerebellar diseases, improvement of gait speed, and activities of daily living (ADLs) via intensive rehabilitation was reported [71,72]. We believe that such plasticity will help the cerebellar reserve in several ways:

(a) Redistribution of synaptic weights. The circuit can redistribute the synaptic weights according to the demand, the constraints, and the complexity of the environment. Though a causal relationship between improvements of CAs via rehabilitation and induction of synaptic plasticity is elusive, it would be plausible that a new internal model of coordinate movement is acquired in relatively intact regions of the cerebellar cortex via rehabilitation training by changing the strength of synaptic transmission. Through the rehabilitation process, a new set of sensory inputs and efferent copies would cause STDP at the input stage of the cerebellar cortex, and a new internal model would be acquired gradually via rebound potentiation of inhibitory synapses onto PCs in the same microzone and via LTP of stellate cell synapse onto a part of PC dendritic branches, and finally LTD at individual PF–PC synapses. It is difficult to obtain direct evidence of such possible synaptic plasticity in patients’ cerebellum. However, the importance of LTD at PF–PC synapses in the improvement of symptoms is strongly suggested in immune-mediated cerebellar ataxias (IMCAs). Some IMCA patients have antibodies against voltage-gated Ca channel (VGCC, P/Q-type), metabotropic glutamate receptor type 1 (mGluR1), and/or glutamate receptor delta (GluR delta). Because these proteins are indispensable for LTD induction, antibodies against these proteins should cause cerebellar ataxia through blocking of LTD. Immunotherapies improved symptoms in IMCA patients having antibodies against these proteins, suggesting that recovery of LTD at PF–PC synapses would be important for the maintenance or acquisition of the internal model of movement [73,74].
(b) New synapse formation. New synapse formation occurs between PFs and PC dendritic spines following intensive training [75]. Synaptogenesis is thus dependent on activity, and the PC spines represent a major site for this phenomenon. Experience-dependent changes of spine structure and number likely contribute to long-term memory storage [76]. Structural spine plasticity in the cerebellar PC is a neurobiological mechanism underlying the acquisition of complex motor skills.
(c) Extra-cerebellar plasticity. When a connection is lost, a substitution mechanism occurs to compensate it. This might occur for instance after a cerebellar stroke or any focal injury in the cerebellar circuitry. The substitution mechanism may include regions outside the cerebellum promoting cerebellar recovery, such as the sensory cortex [77].

## 4. Neuromodulation Therapies That Potentiate Cerebellar Reserve

Rehabilitation is considered to facilitate compensatory reorganization in the central nervous system (CNS). Motor rehabilitation has been proven effective not only in patients with limited lesions but also in patients with degenerative CA [71,72]. At present, non-invasive cerebellar stimulation (NICS) and neurotransplantation are considered promising treatment modalities that could reinforce the cerebellar reserve.

### 4.1. Non-Invasive Cerebellar Stimulation (NICS)

The aim of NICS is to modulate cerebellar controls on the cerebral cortex and the spinal cord, thereby relieving CAs [78]. NICS includes transcranial direct-current stimulation (tDCS), transcranial alternating-current stimulation (tACS), and transcranial magnetic stimulation (TMS). tDCS and tACS tune neural excitability, while TMS induces the generation of action potentials in addition to tuning excitability [78].

Stimulation over the cerebellum using TMS delivered 5–6 ms before the cerebral stimuli reduces the excitability of M1 [79]. This suppression, termed cerebellar brain inhibition (CBI), is explained by PCs (inhibitory neurons)-mediated inhibition of the DN–thalamus–M1 pathway (M1) [79]. It was shown that cathodal tDCS reduced CBI (inhibitory modulations on PCs), whereas anodal tDCS exerted the opposite effect (excitatory modulation on PCs) [80], suggesting that NICS affects the modulation by the cerebellar cortex on cerebellar efferents. A recent physiological study showed that activation of DNs generated by reduced inhibition from PCs (*¡* disinhibition) facilitates the execution of a particular movement, while suppression of the DNs by increased PC activity (i.e., inhibition) contributes to the stabilization of unnecessary movement [30]. Thus, NICS modulates the disinhibition/inhibition mode so as to repair impaired cerebellar output signals and improve CAs. The plasticity of the cerebellar cortex synapses is assumed to be the neural basis for long-lasting modulation [81]. Consistently, animal experiments showed that a hyperpolarization current induced a long-term increase in the firing rate of Golgi neurons [82].

A systematic review by Nuzzo et al. [83] analyzed the therapeutic benefits of NICS on CAs in 170 patients in seven studies (in the three of the seven studies, the effects were examined in a sham-controlled design) [84,85,86,87,88,89,90]. The therapeutic effects were confirmed in a 10 m walk [84,85,87], clinical scores (Scale for the Assessment and Rating of Ataxia or International Cooperative Ataxia Rating Scale) [86,89,90], long-latency stretch reflex [86], and timing of antagonistic commands [88]. On the other hand, the therapeutic value of NICS is limited in the presence of advanced cerebellar atrophy in patients with degenerative diseases [78].

### 4.2. Neurotransplantation

Due to the specific, complex, and elaborate cell-to-cell connections, it is difficult to substitute the damaged CNS region with grafted cells [91,92,93,94]. Not only the grafted stem cells but also the host cells are responsible for the reconstruction of the damaged neural circuits. Cerebellar transplantation comprises two stages: (1) rescue of neurons from extinction in order to maintain the cerebellar reserve and (2) reconstruction of damaged cerebellar circuits for potentiation of the cerebellar reserve [93].

Transplantation provides neurotrophic and/or metabolic support to the degenerating cells, suppresses inflammatory reactions, reduces glial activation, and rectifies specific pathological factors responsible for degeneration, leading to the maintenance of the cerebellar reserve [95,96,97,98,99,100,101]. Mesenchymal stem cells grafted into the cerebellum of newborn Lurcher mice produced neurotrophic factors, such as brain-derived neurotrophic factor (BDNF), neurotrophin 3 (NT-3), and glial-derived neurotrophic factor (GDNF), and rescued Purkinje cells from degeneration [99]. Transplantation of neural stem cells in mice with Machado–Joseph disease was followed by increases in the levels of various neurotrophic factors and reductions in neuroinflammatory reactions [101]. Furthermore, grafted neural stem cells formed gap junctions with host PCs at risk of degeneration in SCA1 mice, thus rescuing them from cell death [97]. Transplantation of bone marrow-derived cells fused with the host’s PCs enhanced the survival of degenerating PCs in PCD mice [90]. Another study reported that grafted neural stem cells reduced excessive levels of tissue plasminogen activator, which is known as a neurotoxic agent in mutant mice [96].

In pathological conditions, the residual cerebellar tissues are susceptible to facilitation processes [93]. BDNF, besides the above cell-protective effects, upregulates glutamatergic vesicles at the PF–PC synapses [102] and facilitates GABAergic synaptic transmission [103], which contributes to synaptic plasticity. This facilitation process is applicable at a stage when various neuronal functions can be manipulated [93].

### 4.3. Cerebellar Reserve-Based Therapeutic Principles

**Figure 4:**
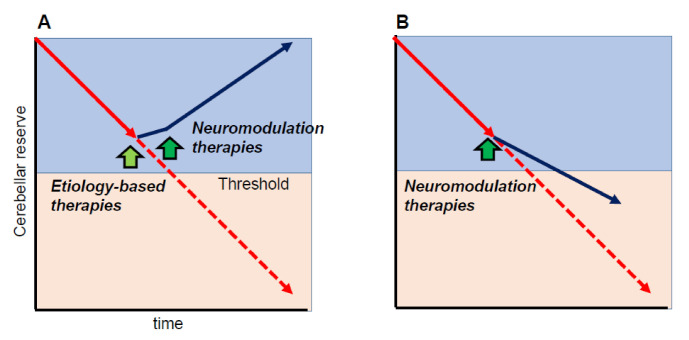
Schematic illustration of the effects of neuromodulation therapies. Neuromodulation therapies can modify cerebellar symptoms if the cerebellar reserve is preserved. (**A**) When etiology-based therapies are available (e.g., abstinence, removal of toxic agents, or diminishment of autoimmune processes,) and disease progression is stopped, neuromodulation therapies should improve cerebellar ataxias (CAs), leading to partial or full recovery of CAs. (**B**) When disease progression cannot be controlled (e.g., degenerative CA), early intervention by neuromodulation therapies can delay disease progression. Yellow-green arrow indicates the timing of etiology-based therapies, whereas green arrows indicate the timing of neuromodulation therapies.

One of the important cornerstones of the cerebellar reserve is that neuromodulation therapies can only be beneficial when the cerebellar reserve is preserved. In other words, neuromodulation therapies can improve cerebellar-related symptoms if the cerebellar reserve is preserved. For example, when etiology-based therapies are applicable (e.g., abstinence, removal of toxic agents, or diminishment of autoimmune processes) and disease progression is halted, neuromodulation therapies will improve CAs, leading to partial or full recovery of CAs (Figure 4A). On the other hand, even if disease progression cannot be controlled (e.g., degenerative CA), early intervention with neuromodulation therapies can delay the progression (Figure 4B).

The combined neuromodulation therapies including motor rehabilitation, NICS, and transplantation might elicit more than additive therapeutic benefits. Furthermore, the activation of endogenous neuromodulators (e.g., serotonin and noradrenaline) can also be combined for enhancement of the reserve [104]. Consistently, serotonin and noradrenaline elicit modulations of synaptic inputs to PCs with a long-term time course [105].

## 5. Conclusions

The cerebellar reserve is a major property of the brain circuitry and needs to be considered for treatment strategies in cerebellar diseases. Potentiation of the inherent restoration and compensation processes provides resilience to the pathology and improvement of CAs, respectively. “Redundant input organization in the cerebro–cerebellum” and “multiple forms of synaptic plasticity” are critical neural mechanisms underlying the cerebellar reserve. In this regard, it needs to be clarified how these mechanisms are potentiated in neuromodulation therapies, including rehabilitation, NICS, and, theoretically, neurotransplantation. The high susceptibility of the cerebellum to functional reshaping represents a major advantage which should be considered to improve our understanding of this unique structure in the brain.

## Data Availability

The concept reviewed in this manuscript is not based on raw data.
